# Addressing institutional and community barriers to development and implementation of community-engaged research through competency-based academic and community training

**DOI:** 10.3389/fpubh.2022.1070475

**Published:** 2023-01-12

**Authors:** C. Claire Hallmark, Krista Bohn, Lance Hallberg, Sharon A. Croisant

**Affiliations:** ^1^The Institute for Translational Sciences, The University of Texas Medical Branch, Galveston, TX, United States; ^2^Sealy Center for Environmental Health and Medicine, The University of Texas Medical Branch, Galveston, TX, United States; ^3^Department of Pharmacology and Toxicology, School of Medicine, The University of Texas Medical Branch, Galveston, TX, United States; ^4^Department of Epidemiology, School of Public and Population Health, The University of Texas Medical Branch, Galveston, TX, United States

**Keywords:** community-engaged research (CEnR), community-based participatory research (CBPR), competency-based training, increasing capacity, education to action

## Abstract

**Introduction:**

The National Center for Advancing Translational Sciences (NCATS) focuses on reducing barriers to effective translational research that rapidly translates science to clinical and community interventions to improve individual and community health. Community-Engaged Research (CEnR) plays a crucial role in this process by bridging gaps between research and practice. It effectively generates bi-directional knowledge and communication by engaging patients and communities throughout the translation research process. Skills development, however, is critical to enable investigators and communities to establish successful partnerships in research. While there are many independent CEnR education programs nationally, few curricula are mapped to identified domains and competencies.

**Assessment of current community engagement educational frameworks and competencies:**

We located three comprehensive efforts to identify CEnR domains and competencies that we aligned to inform development of our curriculum, which we then mapped to these competencies. The first, undertaken by the NCATS Joint Workgroup on Researcher Training and Education and Community Capacity Building (JWG) was developed to assess training opportunities for academic researchers and community partners to increase their capacity to meaningfully engage collaborators in translational research. The JWG identified curricula, resources, tools, strategies, and models for innovative training programs and community engagement in all stages of research. It also conducted a gap analysis of deficiencies in available resources. Using Competency Mapping, they developed a framework for curriculum mapping that included eight domains, each with two to five competencies of knowledge, attitudes, and skills. The second aligned community-engaged research competencies with online training resources across the CTSA consortium, while the third was focused on Dissemination and Implementation training.

**Actionable recommendations:**

Further informed by a conceptual model to advance health equity, we have adapted and integrated these components into a set of modules designed to educate and empower investigators, trainees, students, and community partners to engage in effective CEnR.

**Discussion:**

This curriculum fills an important gap in our workforce development and helps to meet needs of our community partners. Following program evaluation and validation, we will offer the curriculum for use and further evaluation by other groups interested in using or adapting it for their own programming.

## 1. Introduction

Community-engaged research (CEnR) includes the target community as part of the research, where community is defined as a group of individuals affiliated by geographic proximity, health conditions, or other unifying traits or interests commonly shared (1, 2). Translational and clinical research that incorporates the voices of affected communities increases the likelihood of sustaining successful partnerships, developing and implementing successful interventions, and of disseminating those interventions within the community (3). CEnR provides an insider's perspective often missing from traditional research structures and is recognized as critical in bridging gaps between research and practice, thus enhancing translational results (3). However, to truly engage communities in research in meaningful, ethical, and equitable ways necessitates understanding the relationship of research and researchers to communities and acquiring the skills to enable successful engagement. Additionally, understanding the barriers to practicing effective CEnR is required so that they can be properly addressed to ensure effective CEnR is being practiced and upheld. Researchers and their community partners often need extensive training to be able to solicit and integrate community input effectively. Therefore, we created a model to address overcoming institutional and community barriers preventing successful engagement in addition to a CEnR educational training that will be implemented at our institution, which is based upon current offerings from across the Clinical and Translational Science Award (CTSA) Consortium. The primary objectives of this manuscript are to synthesize three comprehensive efforts to identify a framework for CEnR domains and competencies, align and integrate those identified domains and competencies to inform development of our proposed CEnR training, and to offer a model that assists in breaking institutional and community barriers to achieving effective CEnR. This model integrates recent work on domains and competencies which we aligned to suggested action steps identified by community leaders working with the National Academics of Sciences, Engineering, and Medicine (NASEM), CTSAs, and other institutions. This model was created to help assist in addressing institutional and community barriers that inhibit successful community engagement prior to its start. The purpose of this manuscript is for potential users to adopt or build upon our findings and offerings to enhance their own programming.

To this end, the Joint Workgroup on Researcher Training and Education and Community Capacity Building (JWG) was developed by the National Center for Advancing Translational Sciences (NCATS) to assess training and education opportunities for academic researchers and community partners that would increase their capacity to meaningfully engage collaborators in translational research. The Joint Workgroup identified pragmatic curricula, resources, tools, strategies, and models for innovative education and training programs and community engagement in all stages of research (4). It also compiled a database of existing training curricula and conducted a gap analysis of deficiencies in available resources. They then engaged in a comprehensive Modified Delphi Technique for CEnR Curricula Competency Mapping, which resulted in a final framework for curriculum mapping that included eight domains, each with two to five competencies of knowledge, attitudes, and skills (4). Through competency mapping, the committee identified the strongest and most comprehensive CEnR curricula as well as those that require strengthening. The Workgroup identified emphasis on the following competencies: community and stakeholder engagement, cultural and population diversity, translational teamwork and partnerships, cross-disciplinary training, and scientific and collaborative communication. They also identified relative deficits in the competency domains of leadership, regulatory support and knowledge, and ethics and responsible conduct of research.

In addition to the domain competency deficits identified by the JWG in their gap analysis, further barriers to competency-based community-engaged research were discussed in *Principles of Community Engagement*, developed by a task force that included members from the CTSA Consortium's Community Engagement Committee, the National Institutes of Health, Agency for Toxic Substances and Disease Registry, and Centers for Disease Control and Prevention. The five identified barriers included: “engaging and maintaining community involvement; overcoming differences between and among academics and the community; working with nontraditional communities; initiating a project with a community and developing a community advisory board; and overcoming competing priorities and institutional differences” (5). Furthermore, a qualitative study conducted by North Carolina Translational and Clinical Sciences Institute, the CTSA institution at the University of North Carolina at Chapel Hill (UNC) identified five fiscal and administrative barriers and facilitators to conducting community-engaged clinical and translational research. Those barriers are as follows: “level of partnership equity; partnership collaboration and communication; institutional policies and procedures; level of familiarity with varying fiscal and administrative processes; and financial management expectations” (6). This CTSA, the task force, and the JWG share a common vision to improve human health by transforming research and teaching environments to enhance the efficiency and quality of clinical and translational research (5, 6). However, operationalizing that vision requires developing an integrated framework suitable for training new generations of translational and clinical researchers interested in participating in community-engaged research (7). Establishing this framework will entail addressing the competency deficits and institutional and community barriers through vetted and appropriate competency-based CEnR training and education.

Although many community-engagement education and training programs have been developed in recent years, few curricula are mapped to identified domains and competencies, and fewer still are designed to address institutional and community barriers to effective training in CEnR. However, many outstanding curricula have been developed that effectively address these competencies. Our Community-Engaged Research Curriculum draws from and builds upon such programs of excellence, including those from the Meharry-Vanderbilt Community Engaged Research Core of the Vanderbilt Institute for Clinical and Translational Research (8); the University of New Mexico's Center for Participatory Research (9); the Tufts Clinical and Translational Science Institute's *Building Your Capacity: Advancing Research through Community Engagement* (10); the Scripps Translational Science Institute's *Toolbox for Conducting Community-Engaged Research* (11); and the University of Arkansas for Medical Sciences Translational Research Institute's *The Community Scientist Academy Toolkit* (12). Our modules are designed to serve our local community by educating and empowering faculty, students, and community partners in community-engaged research. To date, no community-engaged research curriculum has been implemented at the University of Texas Medical Branch that meets the needs of all parties who play a significant role in community-engaged research (e.g., IRB members, academic investigators, and community-based research partners). This curriculum thus fills an important gap in our workforce training offerings. Following program evaluation and validation, we will offer the curriculum for use and further evaluation by our sister research Centers and other groups interested in using or adapting it for their own programs. Moreover, the model we propose later in this manuscript was created to assess identified institutional and community barriers that limit or prevent engaging in successful CEnR. The model represents a process describing how to address these barriers in the development and implementation of CEnR and CEnR trainings. While the model follows the domains and competencies of our specific training identified by the JWG as exemplary, it can be adapted/adopted by other research centers or institutions that wish to strengthen their own programming through use of these suggested action steps. During the development stage of our training, we designed this model to ensure we were taking the necessary action steps to break such barriers. This allowed us to strengthen the design of our CEnR training and identify deficient areas in the training that need further improvement based on both institutional and community needs.

### 1.1. Brief overview of our CEnR training

The intent of our CEnR curriculum is for participants to better understand community-engaged research and to utilize the information provided in the modules to enhance their skills and confidence in CEnR. The curriculum will be offered to all individuals wishing to expand their knowledge on the topic of community-engaged research. The program's ultimate goal is to strengthen clinical and translational research while improving population health and overall quality of life. It also seeks to improve both health services and public health practice and to positively impact community and environmental norms and behaviors. Participants who complete this educational training will leave with improved skills, behaviors, and attitudes toward communities through community empowerment, stronger community-university trust, and a better understanding of community engagement in terms of research and public health. The CEnR Educational Program is a 6-module curriculum that will be covered over the course of 6 weeks delivered in a classroom setting. The curriculum will address eight community-engaged research domains: Community and Stakeholder Engagement; Cultural and Population Diversity; Translational Teamwork and Partnerships; Leadership; Cross-Disciplinary Training; Scientific and Collaborative Communication; Regulatory Support and Knowledge; Ethics and Responsible Conduct of Research. The objectives of each module following and addressing the community-engaged mapping domains can be found in Table 1. Successful completion of the training will entail the following:

- Completion of all six modules.
- Active participation in class discussions and group exercises.
- Completion of required readings, videos, and case studies.
- Completion of pre- and post-test assessments.

**Table 1:** CEnR domains and competencies offered in this CEnR training derived from the NCATS joint workgroup.

**Mapping domains**	**Competencies: Knowledge, attitudes and skills**
1. Community engagement and scientific and collaborative communication	Articulate principles of community and collaborator engagement
	Demonstrate how to engage communities and other partners in research
	Benefits and challenges of community engagement
	Facilitate group discussions, promotion of health and community literacy (i.e., context, needs, values and perspectives of engaged community) and multicultural communications
	Develop and implement a communication plan to share research findings with partners and those impacted by the research
2. Cultural and population diversity	Social determinants of health in individuals and communities
	Cultural competency vs. humility vs. sensitivity
	Health disparities vs. health equity vs. equality of outcomes
	Benefits and challenges of cultural and social variation relating to research
3. Translational teamwork and partnerships	Building and sustaining inter- and/or multi-disciplinary teams
	Advocating for, facilitating and reconciling multiple points of view
	Building and sustaining community and academic partnerships from research teams
4. Leadership and cross-disciplinary training	Identify potential key collaborators to participate in community-academic partnerships to address significant health issues and disparities
	Effectively lead collaborations with academics, communities and other partners
	Recognize institutional and community context of CEnR and partnerships
	Clarify each team members' responsibility through research process
	Develop and manage budget and other resources (e.g., volunteers, meeting space, etc.)
	Formulate study questions and determine outcomes to be assessed
	Advance various models of peer engagement in research (e.g., advisory, employment, formal partnership, etc.) and their value to different phases of translational research spectrum
	Use narrative-based (i.e., qualitative) and numbers-based (quantitative) methods to identify significant health issues
5. Regulatory support and knowledge	Meet expectations for IRB and community review of research projects and process
	Identify proven processes to establish agreements regarding ownership and dissemination
6. Ethics and responsible conduct of research	Understand all ethical dimensions of CEnR and mutual benefit for all research partners and affected community
	Identify approaches and tools to evaluate and improve the collaborative process

However, we also encourage participation in singular modules for those who wish to further their skills in a certain area. Upon completion, participants will have gained a better comprehension of community-engaged research, how to create strong partnerships, their specific role within community-engaged research, and much more including but not limited to skills such as leadership, decision making, accountability, financial responsibilities, and effective communication.

## 2. Assessment of current community engagement educational frameworks and competencies

In developing our curriculum, the competency domains we identified as key were derived from the JWG community-engagement educational framework along with other community-engaged research competencies identified through assessment of online training resources across the CTSA consortium (13), which were further informed by a framework developed for Dissemination and Implementation training (7). We carefully considered these comprehensive efforts, aligned and integrated competencies where suitable, and adapted them for our use to address institutional and clinical barriers for successfully participating in and improving translational research. Such efforts to strengthen preexisting programming and align competencies and goals are consistent with the overall mission of clinical and translational science to improve population health through effective community-engaged research (14).

In delineating our curriculum's domains and competencies, we adapted the JWG domains and competencies but concentrated them to better fit the needs and requirements of our community and institution. We also focused more intently on the three deficient competency domains revealed by the JWG's gap analysis. In our curriculum, two of the three deficient competency domains the JWG identified are addressed in a learning module devoted to regulatory support and knowledge and ethics and the responsible conduct of research. Based upon their connectivity, the remaining deficient competency domain, leadership, was combined with cross-disciplinary training. Doing so is supported by a study concluding that practical actions on fostering cross-disciplinary research are closely linked to leadership and teamwork that should be planned and implemented at research team and institutional levels (15).

The second comprehensive effort we identified aligned CEnR competencies with online training resources across the CTSA consortium (13). This study cataloged publicly accessible online community-engaged research resources from CTSAs and mapped these available resources to CEnR competency domains (13). They identified eight community-engaged competency domain definitions and characteristics, including: knowledge and perceptions of CEnR; personal traits necessary for CEnR; knowledge and relationships with communities; training of those involved in CEnR; CEnR methods; CEnR program evaluation; resource sharing and communication; and dissemination and advocacy. In aligning these domains with those of the JWG, we adopted a practical approach, i.e., considering the role of particular competencies as they relate to training. Many of the core competency domains were highly correlated with those of the JWG, and where different, we modified our curriculum to reflect both. For example, by definition the identified domain of knowledge and perceptions of CEnR was conceptually close to our own of community engagement and scientific and collaborative communication. Both address the basic principles and concepts integral to understanding and performing community-engaged research (i.e., value of CEnR, history of CEnR, CEnR communication, CEnR approaches). We followed this same process in aligning the rest of our competency domains. One slight deviation was related to the domain of personal traits. While we wholeheartedly agree that personal traits are highly influential in capacity for effective relationship building and partnerships, we chose to focus on development of skills that can be taught, modeled, and learned, while emphasizing the importance of self-evaluation and self-reflection.

The third model we considered was focused primarily upon Dissemination and Implementation training. This conceptual framework identified detailed competencies for researchers participating in community-engaged dissemination and implementation (CEDI) and maps these competencies to domains (7). Shea et al. developed this conceptual framework for CEDI competencies identifying attitudes, knowledge, and behaviors necessary for carrying out the principles of community engagement (7). While mapping their competencies based on the community engagement principles as defined by the National Institutes of Health (NIH), they used a nominal group technique (NGT) approach to determine the competencies for conducting CEDI. They identified 40 competencies mapped to nine domains reflecting the attitudes, knowledge, and behaviors for researchers conducting CEDI research (7). This framework was highly useful in that it contributed content based upon a researcher's readiness to participate in community-engaged research, a key aspect of the educational process and essential for its success. While the focus on community engagement dissemination and implementation research is a more specific approach than our own, we found that it, too, was quite similar to the domains and competencies identified by the JWG. As with those of the Piasecki et al. (13) model, where there were differences, we incorporated content to address both.

After carefully examining the three comprehensive efforts, we used various methodologies to align and integrate their domains and competencies in establishing our own. We conducted a thematic analysis to analyze the qualitative data each comprehensive effort uncovered in their study. This five-step process included: familiarization, coding, generating common themes, reviewing themes, and defining themes. Following this method, we investigated all CEnR components to detect, analyze, and report repeated patterns found within the three comprehensive efforts observed. This allowed us to map, identify, and condense similar domains and competencies to establish a final framework. This framework intends to reduce redundancies in current literature and bridge gaps in domain competency mapping. Using this approach in aligning CEnR domains and competencies across the CTSA Consortium allowed us to produce contextual, real-world knowledge about the social structures, behaviors, skills, and attitudes required for carrying out effective CEnR. The methodology of the three comprehensive efforts we examined were compiled from interviews, observations, and existing data. As touched on previously, the JWG used a modified Delphi technique to identify deficits in their domain competency mapping. In contrast, the Shea et al. study used a nominal group technique approach to identifying their domains and competencies. After careful review of the processes each effort used, we performed a summary analysis that collates the key domains and competencies of each source. We have taken this approach because the three comprehensive efforts under review have a similar structure. This finally led us to our last strategy of aligning the CEnR domains and competencies of our training, in which we examined word repetition, indigenous categories, key words in contexts, and used a compare and contrast approach to determine similarities and differences in related themes. After aligning the three efforts, our final framework was devised and can be observed in Table 1. Table 1 represents the domains and competencies offered in our CEnR training that were adopted from the JWG and identified as exemplary. The only modifications made to these domains and competencies, besides condensing them to meet needs of our institution, were concentrating on the domain deficits the JWG identified and revealed in their gap analysis. To ensure that we focused more intensely on these domain deficits and further address them in our training, we aligned similar domain themes identified by the other comprehensive efforts. The final resource we used to inform our curriculum was the National Academies of Sciences, Engineering, and Medicine's (NASEM) Leadership Consortium: Collaboration for a Value and Science-Driven Health System (2). This group created a conceptual model to advance health equity through transformed systems for health. This model identifies concepts and metrics that can be used to assess the extent, process, and impact of community engagement and also illustrates the dynamic relationship between health equity and health system transformation. The model further examines opportunities to assess community engagement and the potential impact it could have on health and healthcare policies, including factors such as inclusion, diversity, and health equity (2). For this reason, we used this framework to inform meaningful community engagement in our curriculum but modified it to appropriately address the institutional and community barriers to developing and implementing effective community-engaged research training. We followed the same process and methodology in designing this model, focusing on the eight foundational standards the NASEM Leadership Consortium identified:

- Define what should be measured in meaningful community engagement, not what is currently measured.
- Be sufficiently flexible to measure engagement in any community.
- Define health holistically.
- Allow the community to see itself in or identify with the language, definitions, and context.
- Embed equity throughout the model.
- Emphasize outcomes of meaningful community engagement.
- Present a range of outcome options for various stakeholders.
- Communicate the dynamic and transformative nature of engagement.

**Figure 1:**
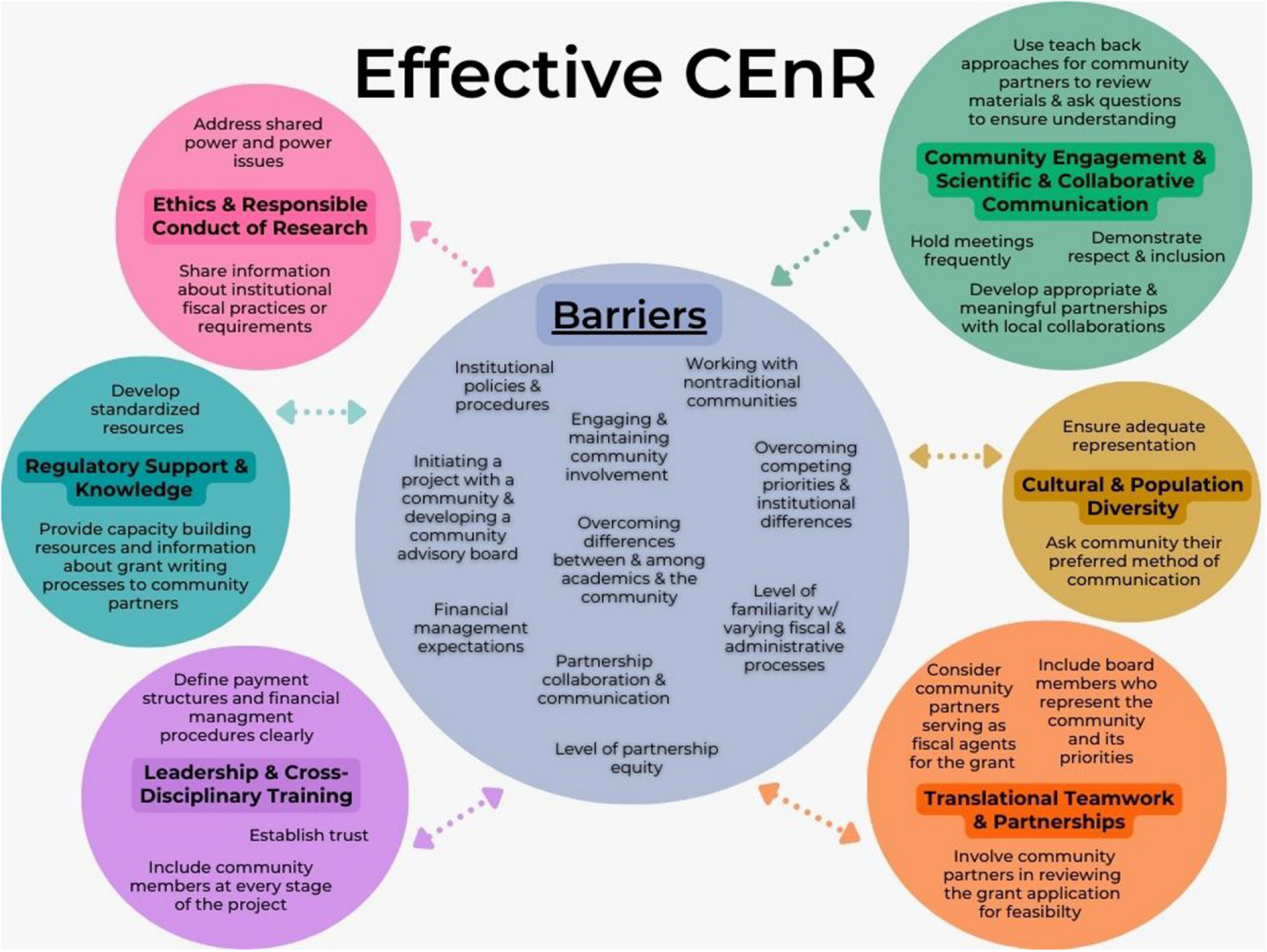
Breaking institutional and community barriers to achieve effective CEnR.

The NASEM conceptual model and our model are similar in that they both address meaningful community engagement; however, the NASEM model is designed to advance health equity through transformed systems for health, whereas our model addresses action steps to breaking institutional and community barriers to effectively develop and implement successful community-engaged research training. As observed in Figure 1, our model centers around the five community and institutional barriers identified in the *Principles of Community Engagement* (5) and the five fiscal and administrative barriers and facilitators identified by the CTSA institution at UNC at Chapel Hill. The model centers around these 10 community and institutional barriers that prevent achieving effective CEnR. From there the model branches off into six circles, each containing our identified domains (i.e., community engagement and scientific and collaborative communication; cultural and population diversity; translational teamwork and partnerships; leadership and cross-disciplinary training; regulatory support and knowledge; and ethics and responsible conduct of research) that were obtained from the JWG's domain competency mapping. These six domains are individually addressed in our community-engaged research training, each making up one learning module. Each circle consists of one of the six domains our curriculum addresses, and within each circle are action steps, each consisting of two to four proposals to help break these institutional and community barriers. The action steps have been aligned with the six domains and their mapped competencies in order of presentation during training. We used a categorization methodology to align these action steps to the objectives of each domain. These action steps have been integrated in our training through various examples and through the content each learning module contains. We conducted an analysis of the qualitative information in which the modules include and differentiated them by certain classes. By accounting for these community and institutional barriers to implementation and development of CEnR training, we hope to ensure the practice of successful and effective CEnR, thus improving translational science and population health outcome. We find these identified action steps essential to breaking institutional and community barriers, and successfully implementing community-engaged research training. The model and recommended actions steps are further addressed, synthesized, and explained in the next section of this manuscript.

## 3. Actionable recommendations

In addressing barriers to effective CEnR and CEnR training, to successfully engage in effective CEnR necessitates having a strong understanding of the institutional and community barriers that prevent successful engagement from occurring. Without addressing these impeding barriers, acquiring the necessary skills, behaviors, and attitudes required for effective participation in CEnR may not be attainable. To this end, we created a conceptual model that demonstrates a process of executing specific action steps to overcome these barriers hindering successful community engagement. The key contribution of our model is to provide emphasis on identified community and institutional barriers. To our knowledge, no model exists that demonstrates a process to break such barriers while integrating CEnR domains and competencies for training. While the model follows the domains and competencies of our specific training identified by the JWG as exemplary, it can be adapted by other research centers or institutions that wish to strengthen their own programming by adopting these suggested action steps and applying them to their own domains and competencies. The model presented was created to help facilitate our CEnR training in the development and evaluation of our domains and competencies. As previously mentioned, the barriers addressed in this model stem from the five community and institutional barriers identified in the *Principles of Community Engagement* (5) and the five fiscal and administrative barriers and facilitators identified by the CTSA institution at UNC at Chapel Hill. We combined and included these ten barriers because we believe each barrier is unique and an important impediment contributing to preventing successful CEnR. The 10 barriers to effective CEnR and CEnR training in this model include (5, 6):

- Engaging and maintaining community involvement.
- Overcoming differences between and among academics and the community.
- Working with nontraditional communities.
- Initiating a project with a community and developing a community advisory board.
- Overcoming competing priorities and institutional differences.
- Level of partnership equity.
- Partnership collaboration and communication.
- Institutional policies and procedures.
- Level of familiarity with varying fiscal and administrative processes.
- Financial management expectations.

To better understand our proposed model, the barriers, and the suggested action steps to break these barriers aligned to CEnR domains and competencies, we created a table (Table 2) that provides firsthand examples as to where in our CEnR training these barriers have been addressed and the necessary action steps to address them in doing so. The intent of this conceptual model is to highlight newly discovered barriers while integrating recent work conducted by the JWG and other CSTAs on CEnR domains and competencies. The major distinction between the conceptual model and the table is that the conceptual model demonstrates the necessary action steps required to address these identified barriers found within each domain, while, in contrast, the table describes how our training accounts for these barriers and where in our training it addresses how to overcome them. For instance, to address the barrier of meeting “financial management expectations,” which the domain of “leadership and cross-disciplinary training” addresses, the suggested action steps to overcome this barrier would be to define payment structures and financial management procedures clearly and ensure strong financial involvement of principal investigators. To achieve these action steps while also breaking this barrier, in our training we describe the process of developing a budget, including an example budget, and have incorporated community member compensation guidelines that the CTSA Collaboration/Engagement Domain Task Force identified. Without describing and providing these examples within our training modules, addressing these institutional and community barriers cannot be done successfully. The suggested action steps can only be done if the content in the modules demonstrates how to accomplish achieving this. In addition to using a categorization methodology to align our domains to these 10 institutional and community barriers, we also independently reviewed and analyzed them using a Rapid Assessment Process (RAP). We use this approach to facilitate key themes among their corresponding competencies and objectives. We chose this approach because of time-sensitivity and because the topic of CEnR is rapidly evolving.

**Table 2:** Addressing institutional and community barriers to achieve effective CEnR.

**Barrier**	**Mapped domain**	**Action steps**	**Examples in training**
Engaging and maintaining community involvement	Community engagement and scientific and collaborative communication	Develop appropriate and meaningful partnerships with local collaborations; Demonstrate respect and inclusion	The nine principles of community engagement; timeline of project; scope of project
Overcoming differences between and among academics and the community	Leadership and cross-disciplinary training	Include community members at every stage of the project; establish trust	Bridging the gap between academia and community; team members responsibility planning
Working with nontraditional communities	Cultural and population diversity	Ensure adequate representation; ask community their preferred method of communication	Communication planning; diversity and inclusion measures; Social Determinants of Health
Initiating a project with a community and developing a community advisory board	Translational teamwork and partnerships	Include board members who represent the community and its priorities	Guidelines for collaboration, 5-step process for initiating CEnR project; logic model; advisory
Overcoming competing priorities and institutional differences	Ethics and responsible conduct of research	Address shared power and power issues	Organizational chart of designated roles; managing expectations; IRB review
Level of partnership equity	Translational teamwork and partnerships	Involve community partners in reviewing the grant application for feasibility; consider community partners serving as fiscal agents for the grant	Sharing in decision making; involvement in grant writing; shared data agreement
Partnership collaboration and communication	Community engagement and scientific and collaborative communication	Use teach back approaches for community partners to review materials and ask questions to ensure understanding; hold meetings frequently	Memorandum of Understanding; timelines; roles
Institutional policies and procedures	Ethics and responsible conduct of research	Share information about institutional fiscal practices or requirements	Review of institutional policies regarding research; procedure plan
Level of familiarity with varying fiscal and administrative processes	Regulatory support and knowledge	Develop standardized resources; provide capacity building resources and information about the grant writing process to community partners	Community partner toolkits; list of requirements; technical support network for administrators
Financial management expectations	Leadership and Cross-Disciplinary Training	Define payment structures and financial management procedures clearly; ensure strong financial involvement of principal investigators	Developing budget; community member compensation guidelines

Prior to creating our community-engaged research training, we examined the suggested recommendations for future improvements from the models we adapted. Suggestions included improving efforts across the CTSA consortium for navigating community-engaged research online information, materials, and resources; increasing access to CTSAs' publicly online CEnR coursework; and establishing standardized nomenclature. Other recommendations include establishing a gold standard for community-engaged research for CEnR domains and competencies across the CTSA consortium while reducing redundancies (13). In aligning these frameworks to build our own, we experienced this challenge firsthand. While we have established a program that will work well for our purposes and will hopefully be of use to others, it is not and was not intended to be a gold standard. However, development of a true gold standard remains necessary to optimize an effective community-engaged research training that could be widely adopted across CTSAs and other similar research entities with a need for effective CEnR training. This will ensure consistency across programs and that trainees completing programming will have similar abilities in taking the field.

Shea et al. suggested developing a community-engaged dissemination and implementation/ community-engaged (CEDI/CE) research readiness survey, based on their proposed domains and competencies (7). This readiness survey is to accurately measure a researcher's attitudes, willingness, and self-reported ability for acquiring the knowledge and performing the actions necessary for effective community engagement (7). We found their framework highly useful in building and designing our curriculum. We adapted their suggestion to employ a community-engaged research readiness survey into our pre-and-posttest assessments to gauge and evaluate all participants' knowledge and readiness in practicing effective community-engaged research. They also emphasized the importance of training on how to establish and maintain effective research/community partnerships and included a level of granularity in their competencies that was lacking in other models. We were able to include specific suggestions throughout our curriculum, particularly related to capacity and relationship building.

The JWG Domain Task Force's final report for NCATS identified four recommendations for further work and research. The four recommendations include (4):

1. Promote access to existing curricula, support the maintenance of the community-engaged research curricula inventory, and ongoing availability of resources provide curricula accessibility through online, searchable platforms.
2. Use the identified curricular gaps from the JWG's gap analysis, those deficits being the competency domains of leadership, regulatory support and knowledge, and ethics and responsible conduct of research, to encourage the development of additional curricula that addresses these deficiencies.
3. Encourage NCATS to disseminate the JWG report and its findings extensively.
4. Encourage stakeholders and community partners to collaborate with the CTSA hubs to develop new programs addressing the diverse and changing needs of community partners and academic researchers.

Based upon the models and their suggestions for future improvement, we developed our own CEnR framework and selected the supporting curriculum, which is geared toward a broad-based audience including investigators, students, trainees, and community partners. We mapped our curriculum to the domains and competencies in a series of six modules that will be covered over the course of 6 weeks. The training addresses the eight community-engaged research domains identified by the JWG, supplemented by additional competencies included in the Shea et al. (7) and Piasecki et al. (13) models. The eight domains include: Community Engagement; Cultural and Population Diversity; Translational Teamwork and Partnerships; Leadership; Cross-Disciplinary Training; Scientific and Collaborative Communication; Regulatory Support and Knowledge; Ethics and Responsible Conduct of Research. The domains are covered in the six modules, each which provides training in two to eight competencies (see Table 1). Within the curriculum, the competency domains of leadership, regulatory support and knowledge, and ethics and responsible conduct of research have been further addressed, defined, and developed to fill this competency gap.

## 4. Discussion

Community-engaged research and supporting training efforts are greatly increasing, providing opportunities for increased collaborations across CTSA hubs and other research institution partners in these endeavors. Current independent CEnR education programs and educational offerings propose conceptual frameworks that identify the components and principles of community-engaged research, but few are mapped to identified domains and competencies or focus on addressing institutional and community barriers. Although some frameworks and trainings are available for building and assessing partnerships between community collaborators and researchers, few describe a process for translating research into practice (7). In addition, the available community-engaged research domains and competencies identified by the JWG and other online training resources across the CTSA consortium must be further tailored to the diverse and changing needs of academic researchers and community partners (4). The University of Texas Medical Branch has not previously implemented a community-engaged research curriculum that primarily focuses on all parties involved in community-engaged research (i.e., investigators, trainees, students, and community partners). Thus, this CEnR curriculum fills an important gap in our workforce and helps meet the needs of multiple community partners and investigators. This curriculum not only identifies all facets of successful community-engaged research and provides a process for translating research into practice, but also outlines the attitudes, knowledge, and skills required for effective CEnR. The content of this training focuses on the research process, community involvement, sustainability of partnerships, research ethics, study design, budget and grant funding, communication planning, and skills development to establish successful collaborations in translational research. It is intended to simplify participation in research for community members, foster strong partnerships, and provide opportunities for open dialogue between researchers and community members.

The model presented herein was created to address institutional and community barriers to effectively develop and implement successful community-engaged research training. However, this model is only one key component of the work needed to ensure these barriers are being properly addressed and that effective CEnR training takes place. Testing this model is essential for evaluating its effectiveness and understanding the most appropriate context and circumstances for its use. Future work should consider integrating CEnR resources and supporting development of standardized curricula for community-engaged research education and its use in translational science. There is still a pressing need for better access to such resources and navigability remains problematic (13). There is also inadequate publication of CEnR literature and limited data related to the core competencies necessary for successful scholarship in community-engaged research (7, 13). Additionally, extreme variations exist in the methods and quality of literature concerning best practices in community-engaged research training, and further research is needed to establish universally accepted competency domains. Steps should also be taken to ensure the adoption of common definitions and language. For example, in our curriculum we have opted not to use the term *stakeholder*, given its potential to offend some tribes and tribal members and its avoidance is now recommended by the Centers for Disease Control and Prevention as a best practice (18, 19). The next step for this CEnR curriculum is to implement it at the University of Texas Medical Branch following program evaluation and validation. This curriculum will be offered for use and further evaluation by our partners and other groups interested in using or adapting its content in their own educational programming.

### 4.1. Limitations

It is necessary to understand this community-engaged research training is designed and oriented to meet the needs of our desired institution and community. It must also be understood that this curriculum is ongoing and needs to be continuously updated and improved as new evidence and research emerge. It is imperative to note that this community-engaged research curriculum requires further evaluation of the efficacy and acceptability of the curriculum at the individual, community, and institutional levels (16). The future direction of this community-engaged research training primarily focuses on facilitator satisfaction, ease of implementation, and institutional and community adoption/acceptance.

## 5. Conclusion

Optimally, addressing institutional and community barriers to effective CEnR will help advance community-engaged research and CEnR training across the CTSA Consortium. Understanding this complex relationship is key to improving the quality of the clinical and translational research enterprise. To our knowledge, no CEnR training exists that integrates recent work on domains and competencies aligned to specific action steps that aim to break institutional and community barriers. Our findings highlight the importance of equitable processes for establishing the necessary skills, behaviors, and attitudes for effective community engagement. Synthesizing these three comprehensive efforts led us to identifying a framework for CEnR domains and competencies that will help to reduce redundancies in current resources and offerings. We then aligned and integrated the domains and competencies to inform development of our CEnR training. We hope that other users can replicate our work or build upon it using a similar methodology. Lastly, we hope that other research institutions will use our model to help overcome the identified community and institutional barriers hindering successful CEnR. Conceptual models are designed, in part, to be utilized as a guiding instrument for advancing science. Conceptual modeling is a framework that is initially used in research to outline the plan of action or to present an idea or thought (17). When conceptual models are developed in a sensible, logical way, they provide a rigor to the research process (17). To our knowledge, no widely used model exists with a purpose of breaking institutional and community barriers while integrating CEnR domains and competencies for training. As demonstrated in the model we designed to address these barriers, we aligned suggested action steps identified by community engagement leaders from across the country working within the CTSAs, NASEM, and other institutions (see Figure 1). We hope that potential users will adopt or improve it for their own programming. Community-engaged research trainings have the capacity to strengthen scientific and translational research while improving population and community health. However, to maximize their effectiveness and ensure consistency in our national programming, integration of efforts and adoption of common metrics is essential. Therefore, the model presented in this manuscript is a key contribution to understanding how to consider, address, and break these institutional and community barriers preventing effective CEnR.

## Data availability statement

The original contributions presented in the study are included in the article/supplementary material, further inquiries can be directed to the corresponding author.

## Author contributions

CH contributed heavily to the educational curriculum upon which the manuscript is based and to the writing of the manuscript and synthesized the educational frameworks into ours. KB co-authored the manuscript in its entirety, contributing heavily to its framework, construction, and revision. LH as educational program director, critiqued the framework, contributed to the authorship, and editing of the paper. SC was responsible for the concept of the paper and contributed significantly to its writing and editing. All authors contributed to the article and approved the submitted version.
